# Redox Biomarkers and Matrix Remodeling Molecules in Ovarian Cancer

**DOI:** 10.3390/antiox13020200

**Published:** 2024-02-04

**Authors:** Elżbieta Supruniuk, Marta Baczewska, Ewa Żebrowska, Mateusz Maciejczyk, Kamil Klaudiusz Lauko, Patrycja Dajnowicz-Brzezik, Patrycja Milewska, Paweł Knapp, Anna Zalewska, Adrian Chabowski

**Affiliations:** 1 Department of Physiology, Medical University of Bialystok, Mickiewicza 2C Street, 15-222 Bialystok, Poland; ewa.zebrowska@umb.edu.pl (E.Ż.); patrycja.dajnowicz@gmail.com (P.D.-B.); adrian.chabowski@umb.edu.pl (A.C.); 2 Department of Gynecology and Gynecological Oncology, Medical University of Bialystok, Marii Skłodowskiej-Curie 24A Street, 15-276 Bialystok, Poland; marta.baczewska@umb.edu.pl (M.B.); pawel.knapp@umb.edu.pl (P.K.); 3 Department of Hygiene, Epidemiology and Ergonomics, Medical University of Bialystok, Mickiewicza 2C Street, 15-222 Bialystok, Poland; mat.maciejczyk@gmail.com; 4 Students’ Scientific Club ‘Biochemistry of Civilization Diseases’ at the Department of Hygiene, Epidemiology and Ergonomics, Medical University of Bialystok, Mickiewicza 2C Street, 15-222 Bialystok, Poland; kamillauko01@gmail.com; 5 Biobank, Medical University of Bialystok, Waszyngtona 13 Street, 15-269 Bialystok, Poland; patrycja.milewska@umb.edu.pl; 6 University Oncology Center, University Clinical Hospital in Bialystok, Marii Skłodowskiej-Curie 24A Street, 15-276 Bialystok, Poland; 7 Independent Laboratory of Experimental Dentistry, Medical University of Bialystok, Marii Skłodowskiej-Curie 24A, 15-276 Bialystok, Poland; anna.zalewska1@umb.edu.pl

**Keywords:** ovarian neoplasms, oxidative stress, nitrosative stress, inflammation, apoptosis

## Abstract

Ovarian cancer (OC) has emerged as the leading cause of death due to gynecological malignancies among women. Oxidative stress and metalloproteinases (MMPs) have been shown to influence signaling pathways and afflict the progression of carcinogenesis. Therefore, the assessment of matrix-remodeling and oxidative stress intensity can determine the degree of cellular injury and often the severity of redox-mediated chemoresistance. The study group comprised 27 patients with serous OC of which 18% were classified as Federation of Gynecology and Obstetrics (FIGO) stages I/II, while the rest were diagnosed grades III/IV. The control group comprised of 15 ovarian tissue samples. The results were compared with genetic data from The Cancer Genome Atlas. Nitro-oxidative stress, inflammation and apoptosis biomarkers were measured colorimetrically/fluorometrically or via real-time PCR in the primary ovarian tumor and healthy tissue. Stratification of patients according to FIGO stages revealed that high-grade carcinoma exhibited substantial alterations in redox balance, including the accumulation of protein glycoxidation and lipid peroxidation products. TCGA data demonstrated only limited prognostic usefulness of the studied genes. In conclusion, high-grade serous OC is associated with enhanced tissue oxidative/nitrosative stress and macromolecule damage that could not be overridden by the simultaneously augmented measures of antioxidant defense. Therefore, it can be assumed that tumor cells acquire adaptive mechanisms that enable them to withstand the potential toxic effects of elevated reactive oxygen species.

## 1. Introduction

Ovarian cancer (OC) is one of the most lethal gynecological malignancies among women in the world. In 2020 it constituted the third most prevalent gynecological cancer globally with a total of 313,959 new diagnosed cases, while 207,252 new deaths were reported globally [1]. The low 5-year survival rates estimated to only less than half of the diagnosed patients are associated with the lack of detectable preinvasive phase, specific symptoms and early diagnostic biomarkers in OC. As a result, OC is diagnosed at the advanced stages (FIGO stages III-IV) in over 75% of patients when malignancy has spread beyond the ovaries to the peritoneal cavity and upper abdominal organs. In line with that, OC is most often diagnosed in postmenopausal women, although ovarian carcinogenesis may occur in females of all ages [2]. Epithelial OC is the most predominant pathologic subtype (nearly 90% of OC cases) that can arise from serous, mucinous, or endometrioid cells; high-grade serous ovarian cancers (HGSOC) represent the most common type of invasive epithelial OC [3]. The origin and trigger for OC development remain under debate, while most commonly repeated prerequisites are an injury to surface epithelial ovarian cells due to cyclic ovulation and hormonal stimulation of the surface epithelium. It has been also proposed that shedding of cancer cells from the fallopian tube’s epithelium plays an important role in tumorigenesis; those cells are then implanted and trapped on the surface of the ovary to produce ovarian or primary peritoneal carcinomas [4]. The current mainstay of treatment is surgical cytoreduction accompanied by adjuvant chemotherapy, although in most cases recurrence occurs within 18 months and eventually resistance to chemotherapy develops [5]. Therefore, depending on OC type, additional therapies such as targeted treatment (e.g., angiogenesis inhibitors, poly(ADP)-ribose polymerase inhibitors) and immunotherapy show progression-free survival benefits [6].

Multiple of the mechanisms underlying tumorigenesis, ECM remodeling, angiogenesis and resultant metastatic ability depend on the activity of proteinases known as matrix metalloproteases (MMPs). The almost 30 MMPs are principal mediators of alterations observed in the microenvironment during carcinogenesis, having both unique and overlapping functions. In diseased conditions, MMPs are dysregulated to enable adjustments in tumor environment that favor a cancer-supporting matrix, and infiltration of cancer cells to adjacent and remote tissues [7]. While most MMPs promote tumor progression, some of them may exert protective effects in the host, which underpins the lack of success of clinical trials employing a first generation of broad-spectrum MMP inhibitors [8]. The last step in collagen degradation is catalyzed by cytosolic metalloproteinase—prolidase (peptidase D, PEPD) that cleaves dipeptides with proline or hydroxyproline at the C terminus. Pyrroline-5-carboxylate reductase (PYCR) and proline dehydrogenase (PRODH) enzymes responsible for the last step in proline biosynthesis and the first step of its catabolism, respectively, have also been associated with the progression of malignancies [9]. New studies that reinstate the prognostic value of matrix-remodeling mediators may unravel therapeutic options to target specific proteases in OC and hence limit disease progression.

One of the factors influencing the expression and activity of MMPs and their tissue inhibitors (TIMPs) are reactive oxygen and nitrogen species (RONS). The persistent generation of RONS is observed at all stages in the ovulatory cycle being susceptible to hormonal fluctuations. Under physiological conditions, the production of RONS and the antioxidant system are in equilibrium so that the body preserves the required levels of RONS. During follicular growth, RONS formation depends on enhanced steroid production and higher cytochrome P450 activity. At the same time, the release of estradiol from granulosa cells stimulates antioxidative activity, e.g., by increasing catalase expression, to prevent oxidative stress [10]. RONS levels peak during ovulation due to increased luteinizing hormone secretion that causes an increase in inflammatory precursors in the ovary, so that RONS levels act as important ovulation signals that mediate follicular wall rupture. A later decline in RONS concentration is related with a production of estrogens, which exert an antioxidant effect [11]. A crucial role of RONS in the ovarian function manifests in a multitude of ways, including their action as second messengers to control meiosis, cumulus expansion, ovulation, corpus luteum formation and regression as well as progesterone secretion [12]. However, excessive RONS production might overwhelm the antioxidant defense system that consists of enzymatic and non-enzymatic antioxidants. Therefore, repeated ovulation imposes considerable oxidative stress, inflammation, and cytokines to ovarian surface epithelial cells, which may promote the development of ovarian diseases including tumor initiation and progression, as well as the possibility to contribute to therapeutic resistance [13]. In spite of the numerous deleterious effects that excessive amounts of RONS exert, a certain level of oxidative stress is required to start the apoptosis of cancer cells. Accordingly, cancer cells can maintain RONS concentrations at the level supporting tumor phenotype and high proliferation rate, while avoiding RONS thresholds that induce senescence, apoptosis, or ferroptosis [14]. Nevertheless, the overactivated defense against RONS is a leading cause of treatment resistance and poor prognosis in OC [15], which underlines the necessity to limit the amount of RONS in a defined range also in a disease. Temporary literature lacks thorough data associating redox biomarkers with the stages of HGSOC, while the often-measured circulating redox biomarkers do not always mirror the changes in the tissue [16]. A detailed understanding of the pathogenesis of HGSOC should precede the selection of a therapeutic approach or diagnostic methods in order to optimize the survival prognosis for the patients. Therefore, in the present study, we examined the magnitude of matrix-remodeling oxidative stress, inflammation and the potential for cellular damage in primary tumor tissue samples of HGSOC based on the TCGA dataset. We also assessed the predictive value of these parameters using receiver operating characteristic (ROC) and investigated their correlation with the selected characteristic features of the patients.

## 2. Materials and Methods

### 2.1. Patients Enrolled in the Study

The study included patients who were admitted to the University Clinical Hospital in Bialystok were clinically diagnosed with serous ovarian cancer and underwent surgery between 2017 and 2021. The exclusion criteria included other than serous histological type of OC, comorbidities such as diabetes, L-thyroxine intake, hyperlipidemia, other metabolic disorders; eventually, the study cohort included 30 patients. The control group included ovarian tissues obtained from non-oncological patients (15 met the inclusion criteria). None of the patients received any treatment (chemotherapy, radiotherapy, or hormone therapy) before surgery. The primary tumor and control ovarian samples were obtained by the Biobank team at Medical University of Bialystok. All scraps included were divided in pieces and individually snap-frozen in liquid nitrogen and stored at −80 °C thereafter. The study was conducted according to the guidelines in the Declaration of Helsinki and was approved by the Ethics Committee at the Medical University of Bialystok (permission number APK.002.221.2021). Consent has been obtained from each patient or subject after full explanation of the purpose and nature of all procedures used.

### 2.2. Antioxidant Enzymes’ Activities

Catalase (E.C. 1.11.1.6) activity in ovarian tissue was determined spectrophotometrically following the method described by Aebi [17]. This method monitors the reduction in the absorbance at a wavelength of 240 nm due to hydrogen peroxide (H_2_O_2_) decomposition to H_2_O and O_2_. One unit of catalase activity corresponds with the amount of enzyme that breaks down 1 mmol H_2_O_2_ in 1 min.

Superoxide dismutase (SOD, EC 1.15.1.1) activity was assayed based on the ability of SOD to remove superoxide anion (O_2_^•−^) and thus reduce spontaneous autoxidation of adrenaline to the adrenochrome in the alkaline environment (at pH 10.2). Changes in the absorbance were determined spectrophotometrically at a wavelength of 480 nm. It was assumed that a SOD activity unit reflects the amount of enzyme which leads to 50% inhibition of adrenaline autoxidation [18].

Glutathione peroxidase (GPx, (EC 1.11.1.9) was determined colorimetrically based on the conversion of NADPH (the reduced form of nicotinamide adenine dinucleotide phosphate) to NADP^+^, where one millimole of NADPH was catalyzed for one minute by one unit of GPx [19].

### 2.3. Reduced Glutathione (GSH) Content

GSH concentration was determined using a colorimetric method based on the reduction of 5,5′-dithiobis-2-nitrobenzoic acid to 2-nitro-5-mercaptobenzoic acid under the influence of GSH contained in the test sample. The formation rate of 2-nitro-5-mercaptobenzoic acid compound is monitored at 412 nm and is proportional to the concentration of total GSH in the sample [20].

### 2.4. Pro-Oxidant Enzymes

NADPH oxidase (NOX, E.C. 1.6.3.1) activity was quantified by the luminescence method using lucigenin as an electron acceptor [21]. One unit of NOX activity was defined as the amount of the enzyme required to release 1 nmol of O_2_^•−^ per 1 min.

### 2.5. Nitrosative Stress Parameters

The nitric oxide (NO) level was determined indirectly through the measurement of its stable oxidation products, NO_3_^−^ and NO_2_^−^, in a Griess reaction. The produced chromophoric azo product was monitored spectroscopically at 543 nm [22].

The peroxynitrite (ONOO^−^) level was determined by a nitration reaction resulting in nitrophenols [23].

The 3-nitrotyrosine (3-NT) level was determined spectrophotometrically using enzyme-linked immunosorbent assay (ELISA; Immundiagnostik AG; Bensheim, Germany) according to the manufacturer’s instructions.

### 2.6. Protein Glycoxidation Products

The contents of dityrosine, kynurenine and *N*-formylkynurenine were estimated on the basis of their characteristic fluorescence using excitation and emission wavelengths of 330/415 nm (dityrosine), 365/480 nm (kynurenine), 325/434 nm (*N*-formylkynurenine) [24].

The advanced oxidation protein products’ (AOPP) concentration was determined by a colorimetric method using chloramine-T as the standard reference. Tissue samples were pre-diluted in phosphate-buffered saline at a ratio of 1:5, and mixed with potassium iodide and acetic acid. The capacity for iodide oxidation was read at 340 nm [23].

The advanced glycation end-products of proteins’ (AGE) concentration were determined by measuring the fluorescence characteristic of AGE derivatives (350 nm/440 nm). Tissue samples were previously diluted in PBS solution in a ratio of 1:5 [23].

### 2.7. Oxidative Damage of Lipids

Malondialdehyde (MDA) concentration was measured based on the reaction with cold thiobarbituric acid in an acidic pH (10% trichloroacetic acid) at 90–100 °C. The produced pink species exert maximum absorption at 532 nm; 1,1′,3,3′-tetraethoxypropane was used as a standard [25].

The concentration of 4-hydroxynonenal (4-HNE) protein adducts was assessed colorimetrically by a commercial ELISA kit according to the manufacturer’s instructions (Cell Biolabs, Inc., San Diego, CA, USA).

### 2.8. Caspase Activity

The activity of mitochondrial caspase-3 (CAS-3, E.C. 3.4.22.56) was evaluated using Ac-DEVD-pNA (Ac-Asp-Glu-Val-Asp-p-nitroanilide) as a substrate. The amount of chromophore, *p*-nitroaniline (pNA), released by CAS-3 activity was determined at a wavelength of 405 nm [26].

The activity of mitochondrial caspase-9 (CAS-9, E.C. 3.4.22.62) was analyzed fluorometrically using AFC:7-amino-4-(trifluoromethyl)coumarin as a substrate. After cleavage of the substrate by CAS-9, free AFC emitted yellow and green fluorescence (505 nm wavelength) which was analyzed using a fluorometer.

### 2.9. Real-Time PCR Reaction

The mRNA levels of selected genes were assessed by quantitative real-time PCR (qRT-PCR) as we previously described [27]. Briefly, RNA was isolated from tissue samples using a NucleoSpin RNA Plus Kit with RNase-free DNase I treatment (Ambion, Thermo Fisher Scientific, Waltham, MA, USA) and an EvoScript universal cDNA master kit (Roche Molecular Systems, Boston, MA, USA) was used to synthesize cDNA. Next, qRT-PCR was carried out using the LightCycler 96 System with FastStart essential DNA green master (Roche Molecular Systems, Rotkreuz, Switzerland) together with the verification of PCR product specificity by melting curve analysis. Primers sequences are listed in Supplementary Table S1. The mRNA levels of target genes were normalized to β-actin and calculated according to the Pfaffl method [28]. All samples were assayed in duplicate.

### 2.10. Metalloproteinases Activity

The activity of gelatinases was measured in 96-well plates pre-coated with anti-MMP2 (ab92536, Abcam, Cambridge, UK) and MMP9 (ab76003, Abcam, Cambridge, UK) antibodies. The degradation of fluorogenic substrate MCA-Pro-Leu-Gly-Leu-Dpa(Dnp)-Ala-Arg-NH_2_, where MCA is the 7-methoxycoumarin-4-yl)acetate corresponded with MMPs activity. The fluorescence was measured at wavelengths of excitation at 325 nm and emission at 393 nm [29].

### 2.11. TCGA Data Analysis

The RNA-seq clinical and phenotypic data of both the TCGA-OV dataset and ‘TCGA TARGET GTEx’ cohort of the UCSC Toil Recompute Compendium [30] was downloaded by using the R software package “UCSCXenaTools” version 1.4.8 [31]. The RNA-seq data was in the form of RSEM expected counts which were log2(expected_count + 1) transformed. Differential expression of target genes between tumors and normal tissue was performed using TCGA and GTEx RNA-seq data extracted from “TCGA TARGET GTEx” gene expression by UCSC TOIL RNA-seq Recompute. The comparison was made using the Wilcoxon test and then visualized by the R software package “ggpubr” version 0.6.0. Survival curves of target genes mRNA expressions for overall and progression-free survival were made with optimal cutoffs by the R software package “survival” version 3.5–7 and ”survminer” version 0.4.9. By using the TCGA-OV dataset, various comparisons of target gene expression between age categories, tumor stages and tumor grades were made using the Kruskal-Wallis H and Wilcoxon signed-rank test and visualized by the R software package “ggpubr” version 0.6.0. Heatmaps for gene expression were created and visualized by R software packages “pheatmap” version 1.0.12 and “corrplot” version 0.92.

### 2.12. Statistical Analysis

Statistical analyses were performed with R software version 4.3.2 and GraphPad Prism software version 8.2.1 (GraphPad Software, Inc., San Diego, CA, USA). To test whether the collected numerical data were normally distributed and verify homogeneity of variances, the Shapiro-Wilk normality and Levene tests, respectively, were applied. Afterwards, Student’s *t*-test or Mann–Whitney U test was used to compare the differences between the groups. For multiple comparisons, the Kruskal–Wallis test followed by Dunn’s post hoc test was applied. The multiplicity adjusted *p* value was also calculated. Because most of the data was not distributed normally, results are expressed via median and interquartile range as dispersion characteristics. The dependence between tested variables was analyzed based on Spearman’s coefficients. The receiver operating characteristic (ROC) curve was used in evaluating the diagnostic ability of the measured parameters to discriminate the true state of the subjects. Corrected *p*-values lower than 0.05 were considered to be statistically significant.

## 3. Results

### 3.1. Matrix-Remodeling Associated and Redox-Related Gene Expression in TCGA Cohort

**Figure 1:**
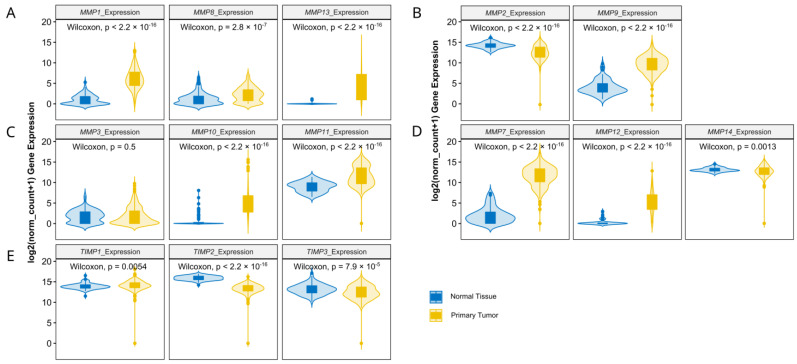
Collagenases (**A**), gelatinases (**B**), stromelysins (**C**), other MMPs (**D**) and TIMPs (**E**) gene expression comparison between TCGA ovarian cancer data and GTEx control group. The number of samples per group were 427 and 88 for the ovarian carcinoma and control tissue, respectively.

**Figure 2:**
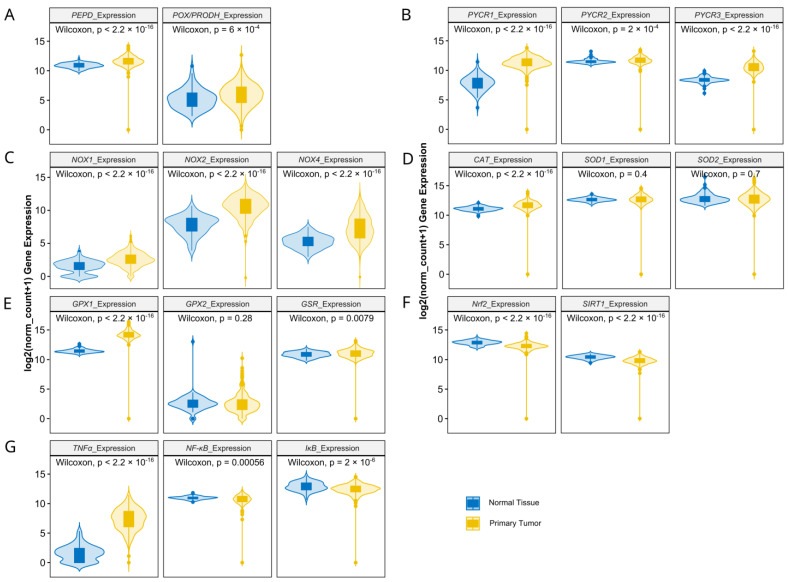
Proline metabolism-related (**A**,**B**), NOXs (**C**), antioxidative (**D**–**F**) and inflammatory (**G**) gene expression comparison by clinical stages of ovarian cancer based on TCGA data. The number of samples per group were 427 and 88 for the ovarian carcinoma and control tissue, respectively.

The analysis of TCGA data confirmed a higher expression level (fold change > 2) of the analyzed MMPs classified as collagenases (*MMP1*, *MMP8* and *MMP13*), stromelysins (*MMP10*, *MMP11*), matrilysins (*MMP7*) and metalloelastase (*MMP12*). Among gelatinases, *MMP2* expression was downregulated, while the *MMP9* level was significantly higher in ovarian cancer. Additionally, stromelysin *MMP3* was unchanged, while membrane-type MMP (*MMP14*) was lower in ovarian cancer as compared to control samples (Figure 1A–D). The expression of TIMPs universally declined in carcinoma tissue (Figure 1E). All the analyzed proline-associated and NOXs genes were enhanced in neoplastic tissue (Figure 2A–C). Among the genes related with antioxidative potential, *CAT* and *GPx1* were enhanced, while *GSR*, *Nrf2* and *SIRT1* upregulated (Figure 2D–F). Similarly, pro-inflammatory gene (*TNFα*) had higher expression, while there were no differences in *NF-κB* and IκB level (Figure 2D).

**Figure 3:**
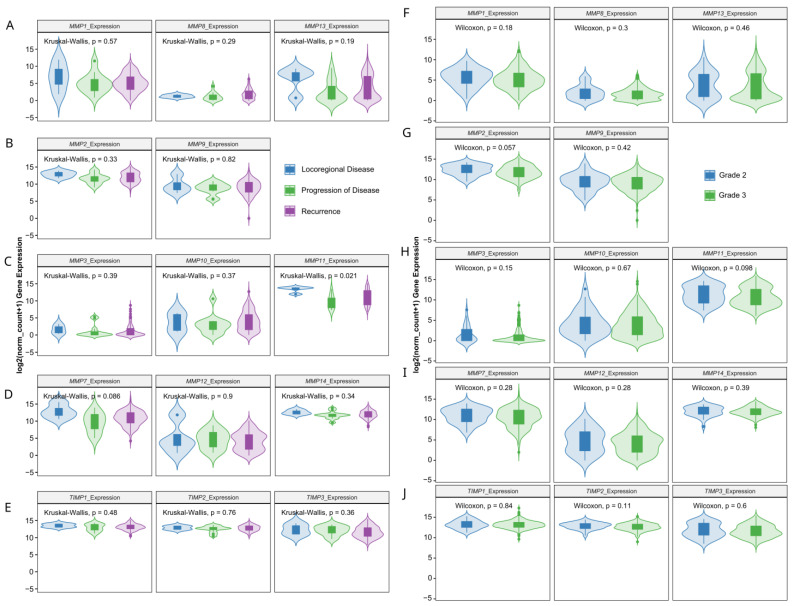
MMPs and TIMPs gene expression comparison in TCGA ovarian cancer dataset by neoplasm type (**A**–**E**) and tumor grade (**F**–**J**). The number of samples per group were 4, 12 and 142 for locoregional disease, progression of disease and recurrence, respectively. Grade 2 cancer encompassed 33 patients, while grade 3 included 260 tissue samples.

**Figure 4:**
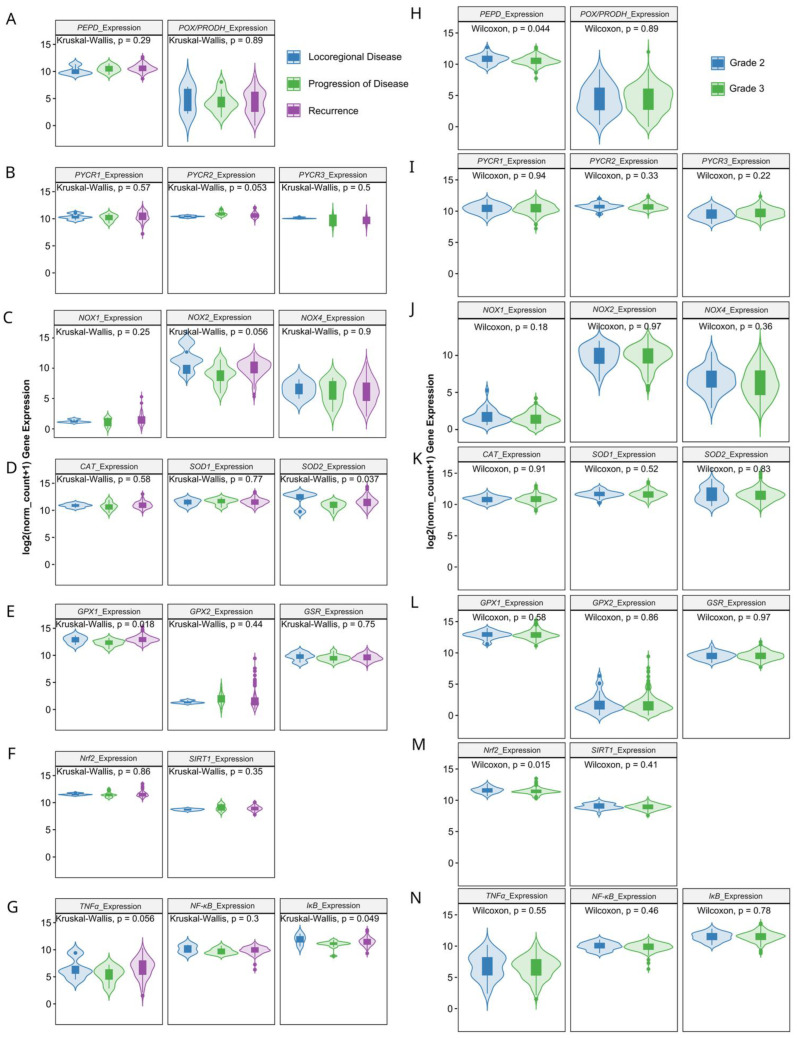
Proline metabolism-related (**A**,**B**,**H**,**I**), NOXs (**C**,**J**), antioxidative (**D**–**F**,**K**–**M**) and inflammatory (**G**,**N**) gene expression comparison in TCGA ovarian cancer dataset by neoplasm type (**A**–**G**) and tumor grade (**H**–**N**). The number of samples per group were 4, 12 and 142 for locoregional disease, progression of disease and recurrence, respectively. Grade 2 cancer encompassed 33 patients, while grade 3 included 260 tissue samples.

**Figure 5:**
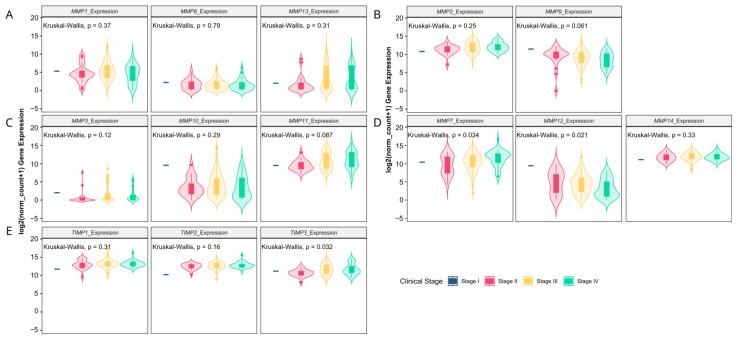
Collagenases (**A**), gelatinases (**B**), stromelysins (**C**), other MMPs (**D**) and TIMPs (**E**) gene expression comparison by clinical stages of ovarian cancer based on TCGA data. The number of samples per group were 1, 21, 241 and 38 for the stages I, II, III and IV, respectively.

**Figure 6:**
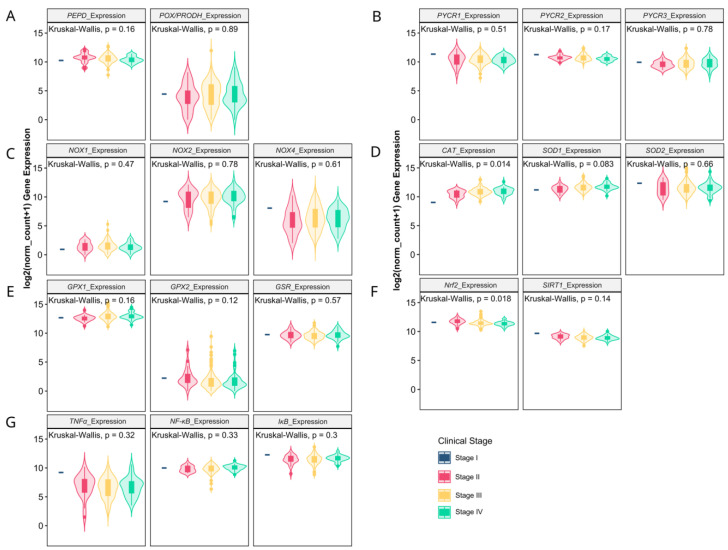
Proline metabolism-related (**A**,**B**), NOXs (**C**), antioxidative (**D**–**F**) and inflammatory (**G**) gene expression comparison by clinical stages of ovarian cancer based on TCGA data. The number of samples per group were 1, 21, 241 and 38 for the stages I, II, III and IV, respectively.

**Figure 7:**
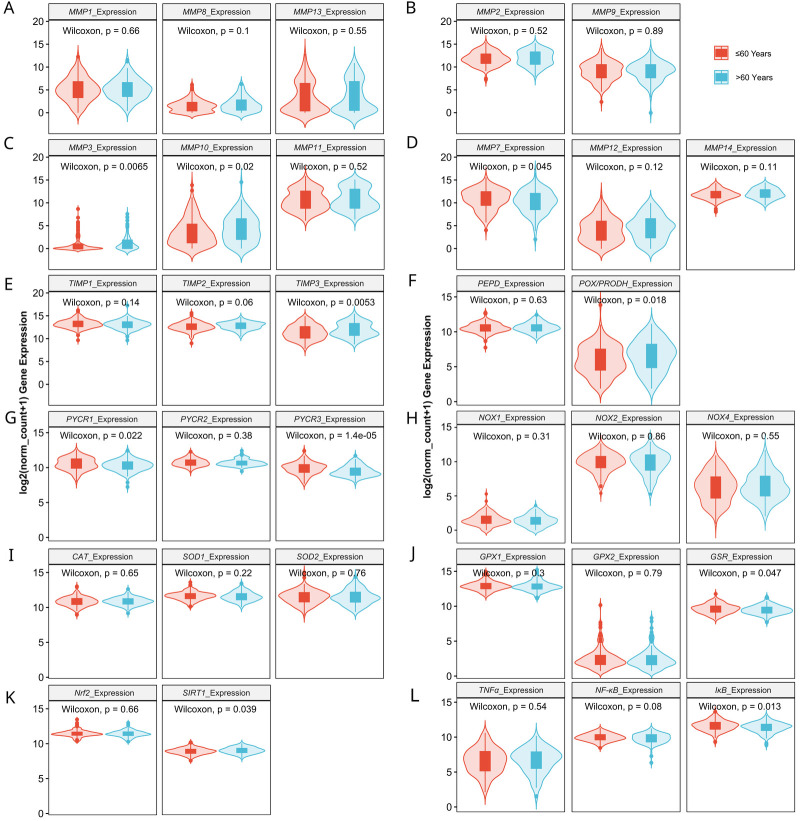
MMPs (**A**–**D**), TIMPs (**E**), proline metabolism-related (**F**,**G**), NOXs (**H**), antioxidative (**I**–**K**) and inflammatory (**L**) gene expression comparison by patients age in ovarian cancer based on TCGA data. The number of samples per group were 175 and 128 for the ages ≤ 60 and >60, respectively.

Next, we verified whether MMPs and oxidative-stress associated gene expression levels correlate with clinicopathologic characteristics. We found that the progression of disease or recurrence were associated only with the lower mRNA level of *MMP-11* (Figure 3A–E), *SOD2*, *GPx1* and *IκB* (Figure 4A–G). *MMPs* did not show significant differences between different OC grades (Figure 3F–J), while *PEPD* and *Nrf2* were significantly lower in grade 3 than grade 2 cancers (Figure 4G–L). Moreover, only the expressions of *MMP7*, *MMP12*, *TIMP3* (Figure 5), *CAT* and *Nrf2* (Figure 6) were affected by the clinical stage of ovarian cancer. An age-associated pattern of gene expression was noticed for *MMP3*, *MMP10*, *MMP7*, *TIMP3*, *POX/PRODH*, *PYCR1*, *PYCR3*, *GSR*, *SIRT1* and *IκB* (Figure 7).

### 3.2. Relationship between Expression Levels of Matrix-Remodeling Associated and Redox-Related Gene Expression in TCGA Cohort

**Figure 8:**
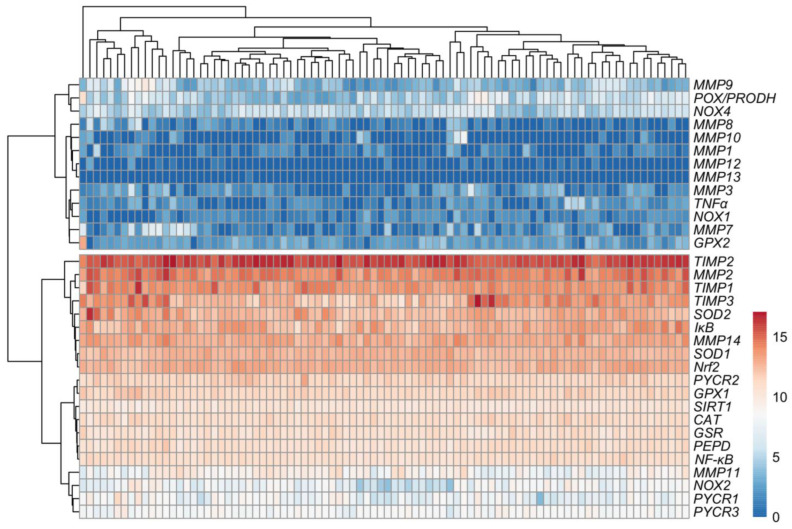
Expression profiles in control samples (*n* = 88) based on GTEx data.

**Figure 9:**
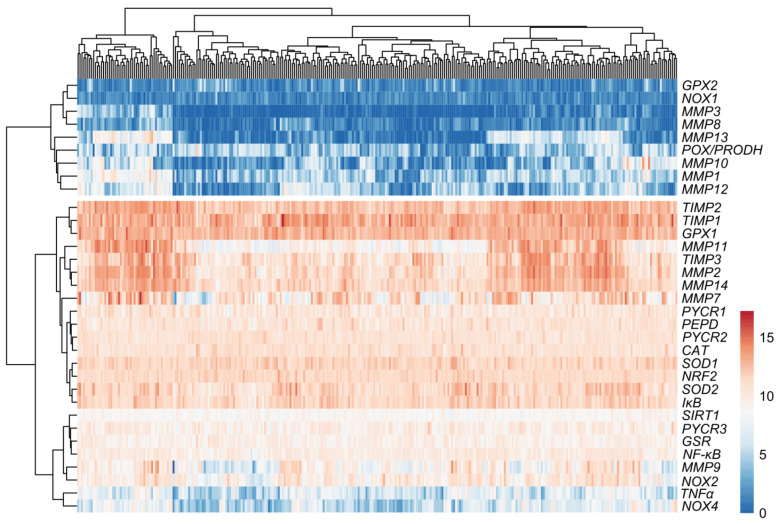
Expression profiles in ovarian cancer samples (*n* = 308) based on TCGA data.

**Figure 10:**
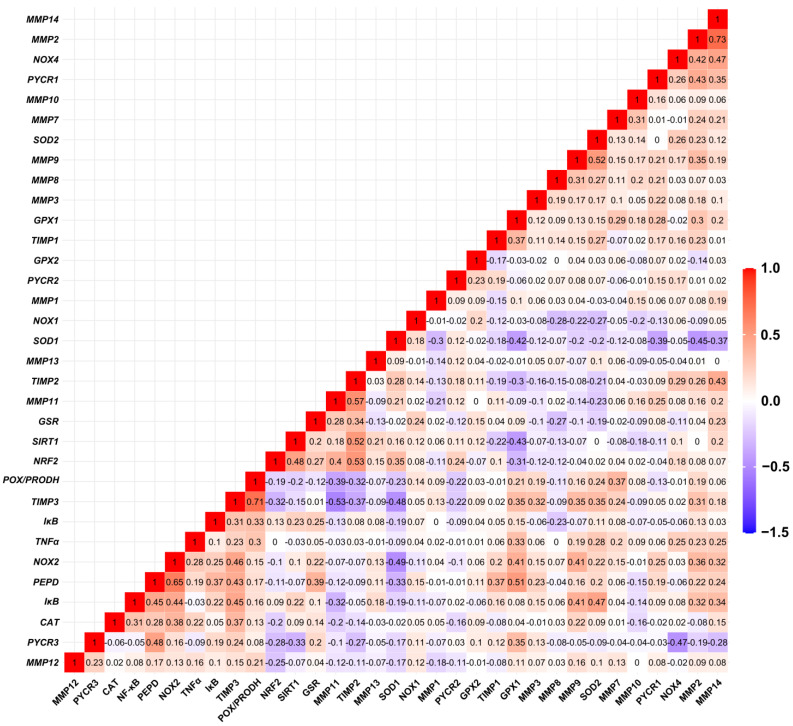
Spearman’s correlation between mRNA levels of selected genes in control ovarian samples based on GTEx data (*n* = 88).

**Figure 11:**
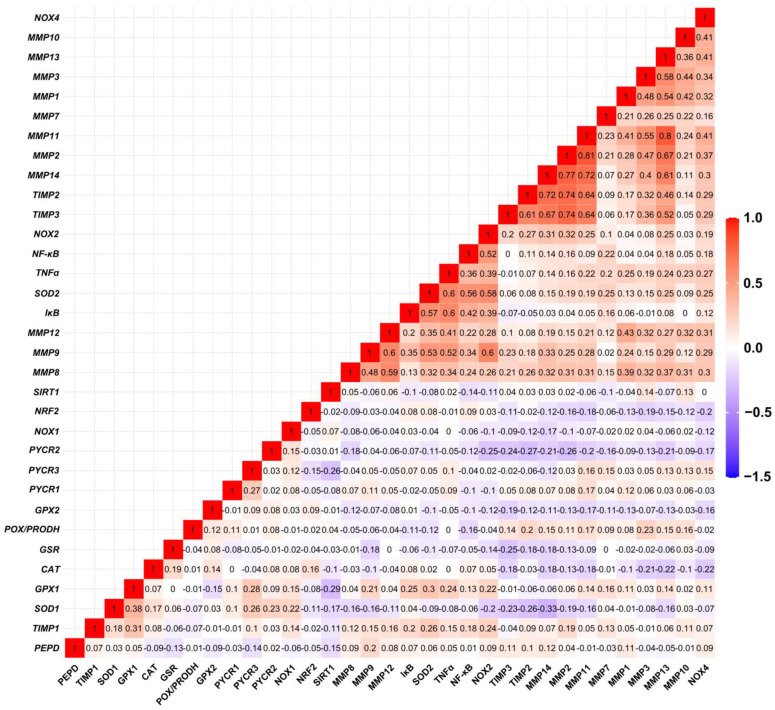
Spearman’s correlation between mRNA levels of selected genes in ovarian carcinoma samples based on TCGA data (*n* = 308).

The analysis of the expression profiles in the ovarian control (Figure 8) and cancer samples (Figure 9) showed varied matrix remodeling- and oxidative stress-related levels among the patients. Generally, TIMPs expression was at higher level than the other genes as compared to the other genes in most cases. Further comparison of the relationship between the genes revealed a negative association between *SOD1* and *MMP2*, *TIMP3*, *PYCR1* and *NOX2* (*p* < 0.05). Among the other oxidative genes, *NOX2* was positively correlated with *TIMP3* and *PEPD*, while *NOX4* with *MMP2*, *MMP14* and *TIMP2* (*p* < 0.05; Figure 10). Contrary to the control, cancer samples exhibited several positive associations between different MMPs and TIMPs to suggest similar patterns of expression regulation in ovarian carcinoma. Among those, both *TIMP2* and *TIMP3* correlated with the level of *MMP2*, *MMP3*, *MMP11*, *MMP13* and *MMP14*. Moreover, *NOX2* was related with *MMP2*, *MMP9* and *MMP14* mRNA level, while *NOX4* with *MMP10* and *MMP13* (*p* < 0.05). The expression of *MMP8*, *MMP9* and *MMP12* was also positively related with *TNFα* level (*p* < 0.05; Figure 11).

### 3.3. Prognostic Value of Matrix-Remodeling Associated and Redox-Related Gene Expression in Ovarian Cancer

**Figure 12:**
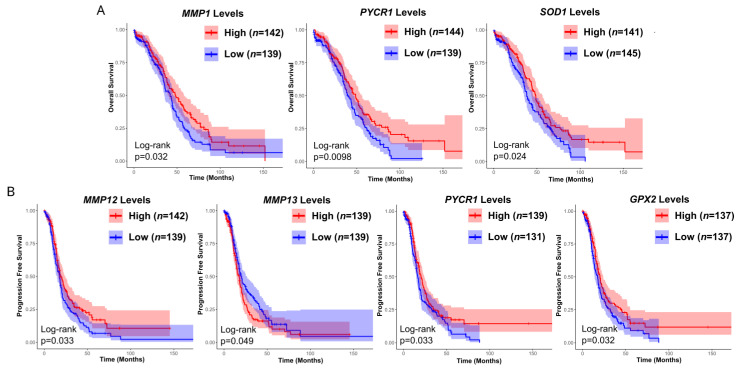
Kaplan-Meier curves of significant overall (**A**) and progression-free survival (**B**) by the matrix- and oxidative stress-associated genes level based on TCGA dataset. OC samples were assigned into two separate groups depending on whether target expression of each sample is higher (high expression) or lower (low expression) than the median. *n*: number of patients.

From all of the analyzed genes, high *MMP1*, *PYCR1* and *SOD1* were associated with a favorable overall prognosis (Figure 12). A hazard ratio lower than 1 corresponding with a lowered risk was noticed for *MMP1*, *PYCR2* and *GPx2*, while values greater than 1 were found for *MMP14* and *TIMP3* (Figures S2 and S3). Progression-free survival depended on the expression level of *MMP12*, *MMP13*, *PYCR1* and *GPx1* in patients with ovarian cancer (Figure 12). Survival was not affected by any other gene (Supplementary Figures S1 and S4).

### 3.4. Patient Characteristics

In the next step, we performed measurements of matrix remodeling-associated genes and redox balance parameters in the samples obtained by the Biobank team at the Medical University of Bialystok. The general clinicopathological characteristics of the control and study groups are presented in Table 1. The study group comprised 30 patients with serous OC, while the proportion of tumors classified as Federation of Gynecology and Obstetrics (FIGO) stages I/II was only 18% (*n* = 5). The remaining patients were diagnosed with grades III/IV (*n* = 25). Based on the phenotype of serous epithelial ovarian cancer, all cancers were classified as grade 3 (high grade). The values of cancer antigen 125 (Ca125) were substantially higher in FIGO III/IV group (+3.76-fold, *p* = 0.0083) as compared to FIGO I/II stages. Based on baseline laboratory examination, the enrolled groups differed by serum platelet count (PLT) and fibrinogen concentration, while PLT also positively correlated with Ca125 level (R = 0.517, *p* = 0.005). These observations resemble previous studies in OC, wherein a rise in PLT was explained by the thrombopoietic cytokine production by tumor and seemed to depend on the amount of tumor cells [32]. Moreover, earlier studies signified PLT as an independent predictor of compromised survival in patients with OC [32]. Furthermore, 13 patients presented extended metastatic lesions at the level of the greater omentum, the so-called ‘omental cake’. The in vivo presentation of HGSOC during cytoreductive surgeries allowed us to distinguish two characteristic subgroups with the preferential invasion of either omentum (*n* = 8) or lymph nodes (*n* = 3).

### 3.5. Matrix Remodeling-Associated and Redox-Related Gene Expression in HGSOC Patients

**Figure 13:**
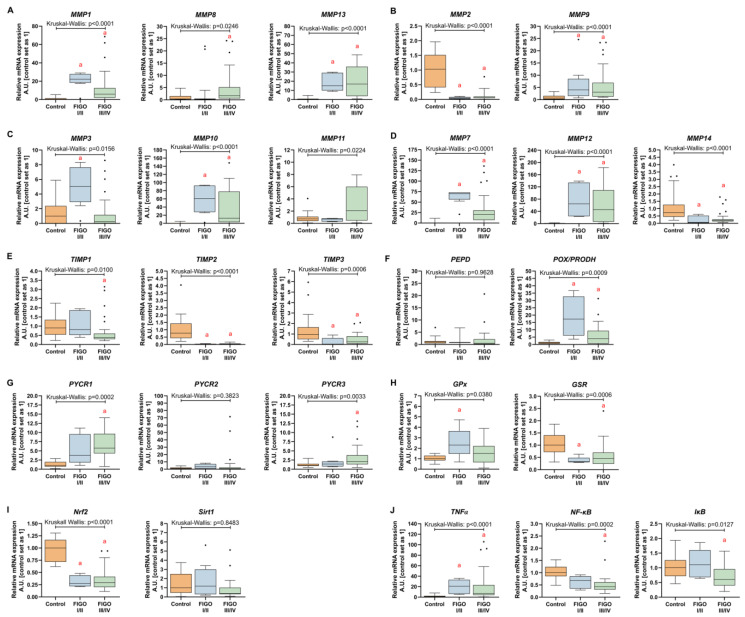
Comparison between matrix-remodeling related genes and redox-associated genes in the control ovarian tissue, and ovarian carcinoma FIGO I/II and FIGO III/IV. (**A**) collagenases (MMP-1, MMP-8, MMP-13); (**B**) gelatinases (MMP-2 and MMP-9); (**C**) stromelysins (MMP-3, MMP-10, MMP-13); (**D**) other MMPs (MMP-7, MMP-12, MMP-14); (**E**) TIMPs; (**F**) prolidase and proline oxidase; (**G**) proline-synthesizing enzymes; (**H**) glutathione metabolism-related genes; (**I**) antioxidative signaling pathways-activating genes; (**J**) inflammatory genes. The number of patients in the control FIGO I/II and FIGO III/IV groups was 15, five and 25, respectively. The results are presented in the form of box–whisker plots with dots to represent the outliers. Significance marker: ‘a’ indicates different vs. control (*p* < 0.05).

Most of the tested metalloproteinases upregulated mRNA expression in both FIGO I/II and FIGO III/IV stages of ovarian cancer (*MMP1*, *MMP13*, *MMP2*, *MMP9*, *MMP10*, *MMP7*, *MMP12* and *MMP14*) indicating profound modifications of the extracellular matrix. The only MMPs with lowered expression in neoplasm tissue were *MMP2* and *MMP14* independently of the cancer grade (Figure 13A–D). The activity of proteases is counteracted by protease inhibitors, including tissue inhibitors of metalloproteinases, TIMPs, which engage MMPs non-covalently to prevent access to their catalytic domain [7]. The expression of TIMPs declined in OC of both stages for *TIMP2* and *TIMP3*, while only in FIGO III/IV in the case of *TIMP1* (Figure 13E). Next, we focused on proline metabolism as a crucial building block of interstitial collagen. The expression of *PEPD* to release proline from exogenous and endogenous proteins was unchanged, but *PRODH/POX* level raised (+16-fold and + 6-fold, FIGO I/II and FIGO III/IV) to suggest proline degradation in mitochondria (Figure 13F). The conversion of glutamine to proline seems to be involved in OC progression since *PYCR1* and *PYCR3* expression was higher in ovarian neoplasm samples from FIGO III/IV stages as compared to the control group (Figure 13G).

Next, we assessed the transcript expression of enzymes involved in glutathione metabolism in HGSOC samples. *GPx* level was substantially elevated in carcinoma tissues of FIGO I/II stages (+1.3-fold), whereas *GSR* expression was diminished independently of FIGO grade (FIGO I/II: −0.67-fold; FIGO III/IV: −0.52-fold; Figure 13H). The expression of nuclear respiratory factor 2 (*Nrf2*), an important controller of an array of antioxidant response element-dependent genes in ovarian cancer, was markedly lower (FIGO I/II: −0.73-fold; FIGO III/IV: −0.67-fold) when compared to the control. We also assessed the expression of *Sirt1*, which has been found to induce antioxidant responses via modulation in SOD2 and catalase, but we did not observe any significant differences between HGSOC and the control (Figure 13I). Furthermore, we observed an increase in the transcript content for *TNFα* (FIGO I/II: +20-fold; FIGO III/IV: +7.7-fold), while the levels of *NF-kB* (−0.56-fold) and *lkB* (−0.40-fold) declined in FIGO III/IV (Figure 13J).

### 3.6. MMPs Activity in Ovarian Cancer

**Figure 14:**
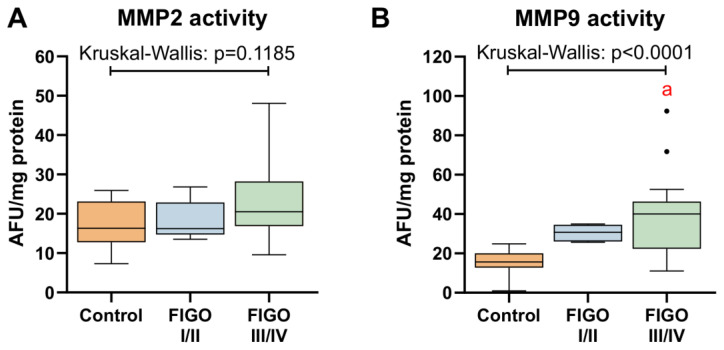
Comparison between (**A**) MMP2 and (**B**) MMP9 activity in control ovarian tissue, and ovarian carcinoma FIGO I/II and FIGO III/IV. The number of patients in the control FIGO I/II and FIGO III/IV groups was 15, five and 22, respectively. The results were presented in the form of box–whisker plots with dots to represent the outliers. Significance markers: ‘a’ indicates different vs. control (*p* < 0.05).

In the next step we verified whether the activity of gelatinases changes with cancer grade. We did not notice significant alterations in the case of MMP2 which corresponds to lower mRNA expression (Figure 14). At the same time, MMP9 activity greatly raised in FIGO III/IV (+159%, *p* < 0.0001), although in FIGO I/II, the change was not significant (+96%, *p* = 0.0931; Figure 14B).

### 3.7. Antioxidant Defense System

**Figure 15:**
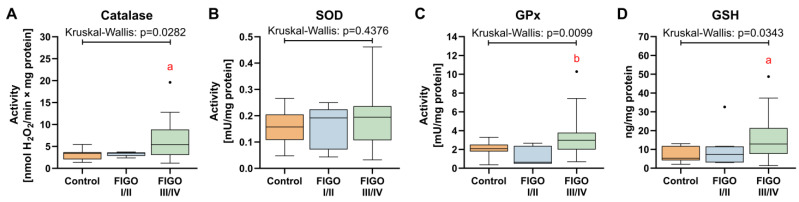
Comparison between biomarkers of antioxidative defense in control ovarian tissue, and ovarian carcinoma FIGO I/II and FIGO III/IV. (**A**) Catalase activity; (**B**) superoxide dismutase activity; (**C**) glutathione peroxidase activity; and (**D**) glutathione concentration. The number of patients in the control FIGO I/II and FIGO III/IV groups was 15, five and 22, respectively. The results were presented in the form of box–whisker plots with dots to represent the outliers. Significance markers: ‘a’ indicates different vs. control (*p* < 0.05) and ‘b’ indicates different vs. FIGO I/II (*p* < 0.05).

Among the studied antioxidant enzymes, only catalase activity was markedly higher (+71%) in advanced grades of HGSOC when compared to the control. There were no considerable differences in SOD activity between the studied groups (Figure 15A). Carcinoma tissues of III/IV grades were also characterized by higher GPx activity as compared to grades I/II (+3.76-fold). Despite that, the content of GSH was markedly increased in advanced HGSOC in comparison to the control (FIGO III/IV: +1.47-fold; Figure 15B).

### 3.8. Markers of Nitrosative Stress and Oxidative Damage in HGSOC

**Figure 16:**
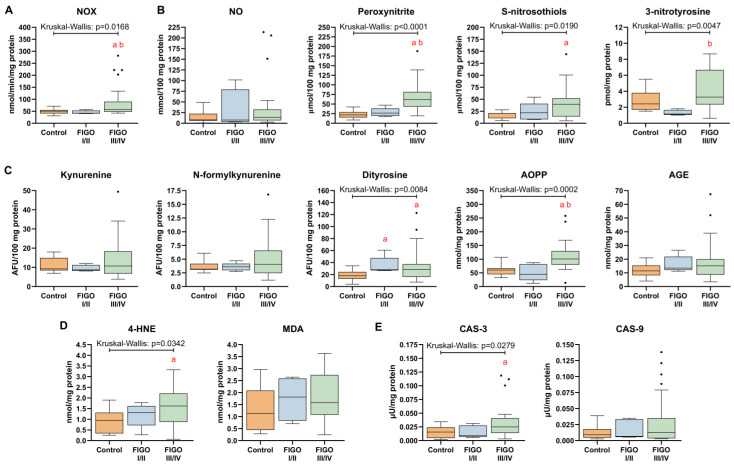
Comparison between pro-oxidative biomarkers in control ovarian tissue, and ovarian carcinoma FIGO I/II and FIGO III/IV. (**A**) NADPH oxidase (NOX) activity; (**B**) indices of nitrosative stress; (**C**) products of protein glycoxidation; (**D**) products of lipid peroxidation; (**E**) caspase 3 and caspase 9 activities. The number of patients in the control FIGO I/II and FIGO III/IV groups was 15, five and 22, respectively. The results were presented in the form of box–whisker plots with dots to represent the outliers. Significance markers: ‘a’ indicates different vs. control (*p* < 0.05) and ‘b’ indicates different vs. FIGO I/II (*p* < 0.05). Abbreviations: 4-HNE: 4-hydroxynonenal; AGE: advanced glycation end-products of proteins; AOPP: advanced oxidation protein products; MDA: malondialdehyde; NO: nitric oxide.

High grades of HGSOC were characterized by an increased NOX activity as compared to both the control (+56%) as well as low-grade tissue samples (+93%; Figure 16A). We did not notice significant alterations in NO content in HGSOC, although peroxynitrite concentration, which is produced by the reaction of NO and superoxide (O_2_^•−^), was elevated in the high-grade HGSOC. Additionally, both analyzed products of nitrosative stress were higher in carcinoma samples, such as S-nitrosothiols (+99% vs. control) and 3-nitrotyrosine (+1.96-fold vs. FIGO I/II; Figure 16B). From the glycoxidation products, dityrosine (FIGO I/II: +47%; FIGO III/IV: +61%) and AOPP (+71% vs. control; +1.3-fold vs. FIGO I/II) content was markedly higher in HGSOC. No significant changes in kynurenine, N-formylkynurenine and AGE specific fluorescence in HGSOC were observed (Figure 15C). The content of one of the lipid peroxidation markers (4-HNE) was significantly increased (+69%) in high-grade HGSOC, whereas MDA content remained unchanged when compared to the control (Figure 16D). Proapoptotic CAS-3 activity was higher in III/IV stages of HGSOC (+84%), whereas CAS-9 activity was similar to the control (Figure 16E).

### 3.9. The Distribution of Redox Biomarkers in HGSOC by BMI, Patient’s Age and Presence of Metastases

Statistical analysis showed that obese patients significantly differed in *MMP3* (−3.33), *Nrf2* expression (+0.934-fold) and *MDA* content (+1.042-fold) from lean individuals, whereas only the *MMP11* level (−2.5-fold) was statistically different between overweight and lean patients. Women at 60 years of age or older had lower levels of *MMP11* (−2.338-fold), *Nrf2* (−0.615-fold), peroxynitrite (−0.751-fold) and CAS-9 (−2.455-fold) as compared to younger patients. NOX was the only parameter to differentiate between women having been pregnant ≥ 2 two times compared to those who had ≤ 1 pregnancies (−1.219-fold). What is more, in metastatic tumors (N1 + N2) primary cancer was characterized by a significantly higher content of MMP2 (+0.660-fold), 3-nitrotyrosine (+0.594-fold), and lower *Nrf2* (−0.600-fold) and *lkB* (−0.954-fold) expressions. Based on in vivo characteristics, tumors categorized as nodal invasion < 50% and ‘omental cake’ exhibited lower MMP7 expression (−0.839-fold), while increased levels of MMP14 (+0.573-fold), TIMP1 (+0.987-fold) and TIMP2 (+1.659-fold). Additionally, higher *Nrf2* (+1.449-fold), 3-nitrotyrosine (+1.096-fold) and *lkB* (+0.759-fold) as well as lower CAS-9 (−1.049-fold) were found when compared to those with > 50% nodal invasion and clear omentum (−1.049-fold; Table 2).

### 3.10. The Analysis of Redox Biomarkers for the Prediction of the HGSOC Occurrence

**Figure 17:**
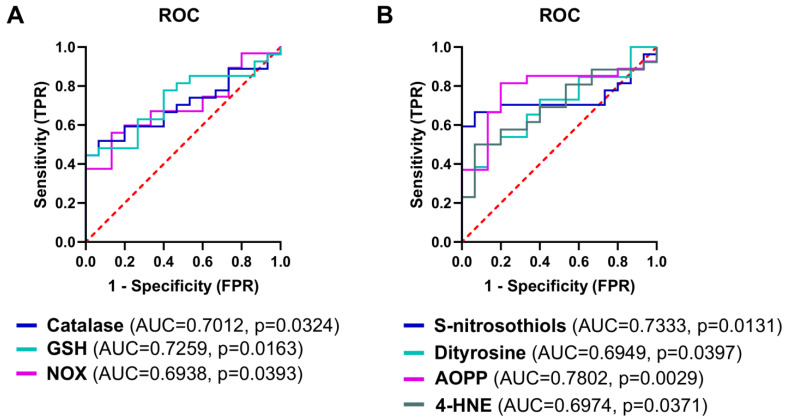
The receiver operating characteristic (ROC) analysis in HGSOC patients for the prediction of HGSOC occurrence. (**A**) The diagnostic value of antioxidant and prooxidant systems. (**B**) The diagnostic value of oxidative/nitrosative damage biomarkers. Only statistically significant changes are included. The number of patients in the control and ovarian cancer groups was 15 and 30, respectively.

The receiver operating characteristic curves were carried out to assess the diagnostic utility of the measured biomarkers in HGSOC occurrence. Among the markers of antioxidant barrier, the highest predictive value was observed for total GSH level (*p* < 0.05; sensitivity = 62.96%, specificity = 60%) followed by catalase activity (*p* < 0.05; sensitivity = 59.26%, specificity = 60%). As for the biomarkers of oxidative damage, the highest discriminatory ability was achieved by AOPP concentration (*p* < 0.05; sensitivity = 80.77%, specificity = 80%; Figure 17). ROC analysis of the assayed biomarkers revealed that none of the redox parameters allows the differentiation between metastatic patients and those without metastasis (Supplementary Table S2).

## 4. Discussion

Signals from the microenvironment, including ECM composition, modulate cancer cell behavior and vastly affect tumor progression. Increased MMPs expression is an early event in ovarian tumorigenesis and is associated with the degradation of epithelial basement membrane and microenvironment reorganization. An imbalance in MMPs and TIMPs expression was found as a negative prognostic factor in hepatocellular carcinoma [33] or gastric carcinoma [34]. In ovarian cancer, the diagnostic usefulness was previously shown for plasma MMP7 and TIMP1 levels in a panel with Ca125 and He4 [35]. Based on TCGA data and our study cohort, the expression of several MMPs rises in ovarian tissue during cancer development together with downstream proline cycle enzymes. We noticed the activation of the proline cycle (↑ *PEPD*, *POX/PRODH* and *PYCR1*) to indicate constant remodeling (synthesis and degradation) of matrix collagen, which in turn can contribute to tumor heterogeneity. High POX/PRODH activity can be considered as a tumor survival factor through ATP production or ROS-induced autophagy [36]. Patients age and tumor morphology were the most confounding factors. For instance, women with a ‘small’ primary tumor, omental invasion and ascites differed in the levels of *MMP7*, *MMP14*, *TIMP1* and *TIMP2* from those with a greater tendency to metastasize to lymph nodes but a clear omentum. In ovarian cancer, peritoneal dissemination is considered to be the most common route for malignant progression [37]. Secondly, the risk of ovarian cancer increased with age in association with *MMP3*, *MMP10*, *MMP7*, *TIMP3*, *POX/PRODH*, *PYCR1*, *PYCR3* to indicate degenerative histological changes. Interestingly, the two gelatinases exhibited differential response to carcinogenesis, which aligns with the absence of co-localization as well as variation in the regulation and functions [38]. *MMP2* is constitutively expressed in various body tissues, while *MMP9* expression depends on several types of transcription factors, such as NF-κB [39]. The meta-analysis data has shown that MMP2 overexpression in ovarian tumor cells contributes to shortened overall survival, but upregulation of stromal MMP2 might be protective [40]. Verma et al. [41] also revealed that MMPs are closely related with oxidative stress. In the TCGA cohort, it was depicted by a correlation between several MMPs mRNA expression in cancer tissue with *NOX2* and *NOX4* transcript level.

A strictly-controlled balance between RONS production and scavenging mechanisms is required for reproductive processes and ovarian homeostasis, however antioxidant barrier capacity progressively declines with age and is followed by the oxidative stress. High RONS concentrations observed at that age are known to induce several pathways for carcinogenesis initiation and progression, including the modulation of cell proliferation, migration, survival, and metabolism. Our results are in line with this phenomena, with an age-related decline in *GSR*, *SIRT1* and *IκB*. An age-dependent rise in RONS level can also be one of the causative factors for late HGSOC diagnosis, which is mostly detected in postmenopausal women, when the impact of oxidative stress accelerates and symptoms become visible. In both humans and animals, reduced age-dependent expression and activity of antioxidant genes was demonstrated, and associated with both ovarian follicle depletion and enhanced oxidative injury to the ovary [42,43]. Overly abundant RONS, exceeding the capacity of antioxidant response mechanisms, can result in significant damage to macromolecules and organelles. However, under chronic exposure to oxidative stress, tumor cells adapt and may become insensitive to RONS-stimulated cell death [44]. In the present study, we observed a concomitant increase in the activity of the main enzymes involved in both the ROS production and removal in cancerous tissue, such as NOX and catalase, respectively, at advanced stages of OC. Circulating antioxidants can also be involved in RONS utilization as evidenced by lowered SOD, CAT, vitamin C and vitamin E levels in the blood of OC patients [45] to suggest a whole-body response to a growing tumor. Additionally, we noticed an increase in GSH content in OC samples, independently of FIGO grade in agreement with the literature data [46]. Since, paradoxically, *GSR* expression was lowered in OC, it suggests that translational and posttranslational changes override the transcriptional decline in GSH synthesizing enzymes in attempt to maintain redox balance. The role of glutathione in ovarian cancer is ambiguous. It might seem that the higher GSH could limit redox imbalance and preclude tumor progression, but clinical studies employing GSH analogue treatments in HGSOC clearly negate this hypothesis [47]. Instead, high GSH content in cancerous tissue has been associated with lower progression-free survival and overall survival compared with patients with low GSH levels [48]. The rise in GSH and glutathione S-transferase P1 (GSTP1) activity, i.e., an enzyme catalyzing the conjugation of GSH to reactive electrophilic compounds, is also noticed during chemotherapy [46] and contributes to reduced cytotoxic effectiveness of cisplatin or carboplatin in human ovarian cancer cell lines [49] and in patients with HGSOC [50].

HGSOC of FIGO III/IV grades exhibited an enhanced level of peroxynitrite, S-nitrosothiols and 3-nitrotyrosine, which is indicative of an uplifted nitrosative stress in advanced stages of the OC. In line with these findings, Li et al. confirmed a positive relationship between high inducible nitric oxide synthases (iNOS) expression with more aggressive phenotypes of HGSOC and poor survival outcome [51]. On the other hand, NOS1 inhibition with NG-nitro-L-arginine methyl ester in ovarian cancer cell lines coincided with diminished cell proliferation, migration and invasion [52]. The subsequent rise in peroxynitrite, a short-lived potent oxidizing agent formed from NO and superoxide (•O_2_^−^), determines oxidative protein and lipid damage [53]. Peroxynitrite may also alter the profile of MHC class I peptides on tumor cells to limit the efficacy of T cell-based cancer immunotherapy [54]. Additionally, NO and NO-derived oxidants mediate some of the major types of protein posttranslational modifications, including S-nitrosylation (reversible modification) and tyrosine nitration (irreversible modification), that affect the key properties of proteins and lead to profound structural and functional consequences and, generally, are used as a biomarkers of intracellular redox state in diseased conditions [55]. The accumulation of 3-nitrotyrosine was associated with metastatic outgrowth, which was also established in human breast carcinoma and resulted in poor prognosis [56]. Whereas the observed herein cellular storage of NO in the form of S-nitrosothiols may reflect an adaptive response of tumor’s cells to counteract the potential pro-apoptotic effects of abnormally accumulated NO and peroxynitrite [57].

As shown previously, high metabolic rates, active inflammation and inadequate tumor neovascularization accelerate RONS generation in HGSOC [44] and can lead to macromolecule damage. In HGSOC tissue, we observed significantly higher levels of early (dityrosine) and late (AOPP) protein glycation products, as well as the product of lipid peroxidation (↑ HNE) compared to the control group. AOPP accumulate by the reaction between chlorinated oxidants (HOCl/OCl^−^) and proteins when oxidation overload is encountered, and reflect both the intensity of oxidative stress and inflammation [58]. In consequence, oxidized proteins may assort structural changes, such as oligomerization, misfolding, and backbone fragmentation, thereby contributing to functional detriment of cells [59]. Most tumor cells develop robust mechanisms in order to tolerate oxidative stress and regulate protein homeostasis, including chaperones, ubiquitin ligases and deubiquitinases. These systems can be a subject of therapeutic intervention, for instance, co-inhibition of GSH synthesis and deubiquitinases was shown to result in the accumulation of polyubiquitinated proteins, induction of proteotoxic stress, and cell death in multiple cancer cell lines [60]. Generally, the primary products of lipoperoxidation, the lipid hydroperoxides, are detoxified, however, when the redox balance is disturbed 4-HNE accumulation occurs as noticed in HGSOC. The exposure to high 4-HNE levels predisposes to the generation of its adducts with amino acyl side chains, such as cysteine, histidine, and lysine residues, via Michael addition as was previously observed in chronic diseases like inflammation, atherosclerosis, and chronic liver diseases [61]. To counteract the toxic effects of 4-HNE (i.e., such as cell growth arrest, mitochondrial dysfunction, apoptosis) tumor cells activate antioxidant defense. Accordingly, a simultaneously lowered 4-HNE concentration and a rise in markers of DNA damage were noticed in breast tumor cells [62]. The differences in 4-HNE content between HGSOC and breast cancer can be attributed to variations in metabolizing capacities, membrane fatty acid composition, or the presence of an accompanying inflammation.

We also noticed that in advanced stages of OC and in primary carcinoma tissues with metastatic potential, RONS can activate NF-κB activity by reducing the expression of its inhibitory protein (IκB) to promote inflammatory response (↑ TNFα). NF-κB augments the expression of genes involved in cell proliferation, apoptosis, and carcinogenesis and further enhances the inflammatory response [63]. The prognostic significance of NF-κB relies on its properties to induce chemoresistance in ovarian cancer cells [64] and to preserve cancer stem cell populations responsible for disease recurrence [65]. The microarray analysis of ovarian RNA transcriptome confirms that both the inflammation- and oxidative stress-related genes can discriminate between young and aging ovaries in a murine model [66]. Additionally, oxidative damage and high levels of inflammatory factors co-exist in several other ovarian disorders, such as premature ovarian insufficiency [67] and ischemia-reperfusion injury [68]. We noticed a higher caspase 3 activity in pretreatment biopsies successively, while in advanced stages of HGSOC there was also an increase in caspase 9 activity. Caspase 3 is vital not only to execute the terminal stages of apoptosis, but also to mobilize progenitor cells and promote tumor regeneration [69,70]. Hu et al. demonstrated that the higher expression of cleaved caspase 3 correlated with clinicopathological markers, such as tumor stage, lymph-node metastasis and differentiation, as well as was predictive of poor prognosis in patients with several cancers of different origin, including gastric cancer, ovarian cancer, cervical cancer and colorectal cancer [71]. It can, therefore, be assumed that caspase 3 stimulates tumor repopulation, hence selecting the best adapted cells and contributing to resistance against treatment. Additionally, it is possible that dying cells release pro-survival factors to promote overall tumor growth [72].

Data from animal models linked obesity with a depleted ovarian reserve and subfertility [73] as well as increased cancer risk due to inefficient DNA repair to process ROS-induced carcinogenesis and with DNA methylation, which alters the expression of genes that suppress tumor progression [74]. Initially, the analysis of the Global Cancer Observatory (GLOBOCAN) database showed an association between OC mortality and metabolic diseases, such as obesity, diabetes and lipid disorders [1]. However, data from the international Ovarian Cancer Association Consortium (OCAC) demonstrated that genetically predicted BMI was associated with an increased risk only for non-high-grade serous subtypes, while the strongest increase was demonstrated for low-grade serous tumors [75]. Similarly, we did not observe a correlation between the tested oxidation markers with BMI, and only MDA and *Nrf2* were substantially increased in obese patients tumors. One potential explanation for higher MDA content is substantially increased lipid content in granulosa cells and the cumulus-oocyte complex, which was confirmed based on experiments on mice fed a high-fat diet [76]. The positive correlation between high-fat diet consumption and *Nrf2* was previously noticed in different tissues of the body [77,78], although the data are not uniform [79,80]. In agreement with our results, the low Nrf2 expression has also been shown to be related with patients’ age [81,82] and serous subtype of OC, whereas its highest level was noticed in the OC of mucinous origin [81].

Some limitations regarding our study should be noted. We did not have information on patients’ diet and physical activity, and we only measured selected biomarkers of nitro-oxidative stress/protein glycation, making it impossible to fully explain the redox impairment in patients with HGSOC. In addition, we only assessed the fluorescence of kynurenine pathway metabolites and not their concentration, which is also a limitation of the study. Recent studies indicate the clinical utility of circulating biomarkers of protein and lipid oxidation/glycoxidation with respect to tumor microenvironment [83], making further research on a larger group of patients in both tissue and blood required.

## 5. Conclusions

**Figure 18:**
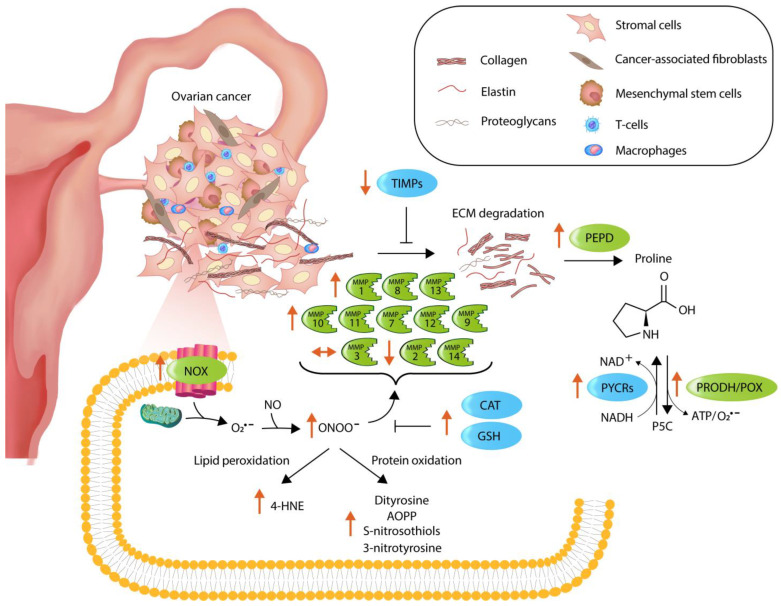
Summary of alterations in matrix remodeling- and oxidative stress-associated markers in ovarian cancer FIGO III/IV. Enzymes accelerating the above processes are in green boxes, whereas blue boxes represent antagonistic molecules. Orange arrows depict how the level of the examined factors has changed in comparison to unaffected ovarian tissue. Black arrows (➝) indicate the direction of process, while ⊢ symbolizes the inhibition.

In summary, an upregulation in matrix-remodeling genes was associated with the level of oxidative stress biomarkers (*NOX2/4*, and *SOD2*), but the prognostic significance of the MMPs, TIMPs and proline-related genes was limited. Moreover, the levels of antioxidants (↑ CAT, GSH), RONS generation (↑ NOX, peroxynitrite), protein modifications (↑ S-nitrosothiols, 3-nitrotyrosine, AOPP), lipid peroxidation (↑ 4-HNE), inflammation (↑ *TNFα*, ↓ *NF-κB*, *IκB*) and apoptosis (↑ CAS-3) are affected by the development of SOC, especially at advanced stages classified as FIGO III/IV. Overall, it can be assumed that the substantial redox imbalance in the high-grade cancer exerts selective pressure on tumor cells to acquire adaptive mechanisms that will enable them to counteract the potential toxic effects of elevated RONS and favor cell survival (Figure 18).

## Figures and Tables

**Table 1:** Study and control group characteristics. Values are presented as mean ± SD. Abbreviations: *n*: number of patients.

	Control	Ovarian Cancer		Control
		FIGO I/II	FIGO III/IV	
Total	*n* = 15	*n* = 5	*n* = 25	-
Age	54.2 ± 11.99	64.2 ± 8.927	61.75 ± 11.05	0.0730
BMI (kg/m^2^)	26.76 ± 3.155	27.96 ± 4.17	28.42 ± 5.49	0.7170
Overweight/obese	*n* = 10	*n* = 3	*n* = 18	-
PLT (×10^3^ cells/mm^3^)	231.1 ± 43.43	247.6 ± 65.21	366.13 ± 113.17	< 0.0001
Fibrinogen (mg/dL)	330.7 ± 54.4	444 ± 51.69	442.1 ± 108.6	0.0019
K+ (mEq/L)	4.203 ± 0.3137	4.778 ± 0.3762	4.616 ± 0.547	0.0073
TSH (µU/mL)	1.572 ± 0.6521	1.731 ± 0.3752	2.024 ± 1.225	0.5790
Glucose (mg/dL)	91.33 ± 9.713	93.6 ± 9.397	94.28 ± 14.96	0.9637
SBP (mmHg)	131.1 ± 17.69	133.8 ± 14.13	141.72 ± 19.22	0.3282
DBP (mmHg)	86.31 ± 8.702	84.2 ± 9.859	82.78 ± 12.29	0.6014
Ca125 (U/mL)	19.06 ± 8.366	243.8 ± 133.5	1161.32 ± 1022.9	0.0083
He4 (U/mL)	35.90 ± 14.00	218.3 ± 172.9	794.4 ± 689.9	0.0409
Primary tumor volume (cm^3^) ^1^	-	767.2 ± 959.6	279.2 ± 488.3	0.0572
Nodal invasion	-	-	*n* = 16	-
Cancer cells in peritoneal fluid	-	-	*n* = 13	-
‘Omental-cake’ ^2^	-	-	*n* = 13	-
Nodal invasion > omental invasion ^3^	-	-	*n* = 3	-
Nodal invasion < omental invasion ^4^	-	-	*n* = 8	-
Time of hospitalization (day)	4.357 ± 1.865	6.8 ± 2.49	10.435 ± 4.99	0.0001

^1^ Calculated using the formula π/6× length × width × height. ^2^ ‘Omental cake’ is a specific term used to describe serious peritoneal disease with a mass-like feature. ^3^ Number of lymph nodes involved > 50% and omentum clear. ^4^ Number of lymph nodes involved < 50% and ‘omental cake’.

**Table 2:** Log2-fold changes in redox biomarkers distributed by the selected characteristic features of all of HGSOC patients. Only parameters with significant changes were included.

	Overweight (*n* = 9) vs. Lean (*n* = 9)	Obese (*n* = 10) vs. Lean (*n* = 9)	Age ≥ 60 (*n* = 19) vs. Age < 60 (*n* = 11)	≥2 Pregnancies (*n* = 9) vs. ≤1 Pregnancy (*n* = 4)	N1 + N2 (Lymph Node Metastasis; *n* = 16) vs. N0 (*n* = 14)	Nodal Invasion < Omental Invasion (*n* = 8) vs. Nodal Invasion > Omental Invasion (*n* = 3)
	Fold Change (*p* Value)					
*MMP2*	0.124 (*p* = 0.905)	−0.030 (*p* = 0.941)	−0.734 (*p* = 0.154)	−0.500 (*p* = 0.799)	0.660 (*p* = 0.050)	1.375 (*p* = 0.235)
*MMP3*	1.141 (*p* = 0.095)	−3.330 (*p* = 0.044)	−1.958 (*p* = 0.973)	−1.050 (*p* = 0.931)	0.440 (*p* = 0.782)	−0.594 (*p* = 0.376)
*MMP7*	0.965 (*p* = 0.661)	−0.132 (*p* = 0.656)	−0.396 (*p* = 0.773)	−1.515 (*p* = 0.874)	−0.476 (*p* = 0.405)	−0.839 (*p* = 0.012)
*MMP11*	−2.498 (*p* = 0.021)	−0.270 (*p* = 0.766)	−2.338 (*p* = 0.012)	0.342 (*p* = 0.620)	1.556 (*p* = 0.115)	0.762 (*p* > 0.999)
*MMP14*	−0.194 (*p* = 0.400)	0.823 (*p* = 0.370)	−1.098 (*p* = 0.318)	0.031 (*p* = 0.642)	−0.176 (*p* = 0.165)	0.573 (*p* = 0.024)
*TIMP1*	−0.066 (*p* = 0.661)	0.668 (*p* = 0.456)	−0.341 (*p* = 0.776)	0.451 (*p* = 0.762)	−0.297 (*p* = 0.515)	0.987 (*p* = 0.024)
*TIMP2*	0.297 (*p* = 0.905)	1.348 (*p* = 0.456)	−1.056 (*p* = 0.559)	0.979 (*p* = 0.292)	0.160 (*p* = 0.209)	1.659 (*p* = 0.012)
*Nrf2*	0.177 (*p* = 0.661)	0.934 (*p* = 0.031)	−0.615 (*p* = 0.042)	0.318 (*p* = 0.504)	−0.600 (*p* = 0.046)	1.449 (*p* = 0.048)
*IκB*	1.087 (*p* = 0.211)	0.819 (*p* = 0.109)	0.538 (*p* = 0.491)	−0.288 (*p* = 0.940)	−0.954 (*p* = 0.043)	0.759 (*p* = 0.049)
NOX	−0.241 (*p* = 0.423)	0.233 (*p* = 0.762)	−0.323 (*p* = 0.074)	−1.219 (*p* = 0.049)	0.016 (*p* = 0.373)	0.535 (*p* = 0.499)
Peroxynitrite	0.127 (*p* = 0.917)	−0.071 (*p* = 0.747)	−0.751 (*p* = 0.223)	−1.634 (*p* = 0.214)	0.198 (*p* = 0.829)	0.631 (*p* = 0.460)
3-nitrotyrosine	0.471 (*p* = 0.645)	−0.083 (*p* = 0.965)	−0.899 (*p* = 0.219)	−0.775 (*p* = 0.497)	0.594 (*p* = 0.036)	1.096 (*p* = 0.048)
MDA	1.342 (*p* = 0.139)	1.042 (*p* = 0.042)	−0.759 (*p* = 0.339)	−0.614 (*p* = 0.683)	−0.318 (*p* = 0.905)	0.877 (*p* = 0.383)
CAS-9	−2.477 (*p* = 0.057)	−1.015 (*p* = 0.633)	−2.455 (*p* = 0.047)	−2.541 (*p* = 0.214)	1.498 (*p* = 0.399)	−1.049 (*p* = 0.036)

## Data Availability

The data presented in this study are available on request from the corresponding author.

## Supplementary Materials

The following supporting information can be downloaded at: https://www.mdpi.com/article/10.3390/antiox13020200/s1, Figure S1: Kaplan-Meier overall survival curves of the matrix- and oxidative stress-associated genes based on TCGA dataset.; Figure S2: MMPs and TIMPs hazard ratio for overall survival in TCGA ovarian cancer patients; Figure S3: Oxidative stress-associated hazard ratio for overall survival in TCGA ovarian cancer patients; Figure S4: Kaplan-Meier progression-free survival curves of the matrix- and oxidative stress-associated genes based on TCGA dataset; Table S1: List of primers used for real-time PCR in the study; Table S2: The receiver operating characteristic (ROC) analysis in HGSOC patients for lymph node metastasis.
