# Supplementary material for: Redox Biomarkers and Matrix Remodeling Molecules in Ovarian Cancer
Source: Antioxidants (Basel). 2024 Feb 4;13(2):200. doi: 10.3390/antiox13020200 (PMC10885995; doi:10.3390/antiox13020200)
Supplement: Supplementary file 1 [file antioxidants-13-00200-s001.zip › antioxidants-2795591-supplementary.pdf]

Supplementary data

Could redox biomarkers be useful in patients with high-grade ovarian cancer?

Elżbieta Supruniuk, Marta Baczewska, Ewa Żebrowska, Mateusz Maciejczyk, Kamil Klaudiusz Lauko, Patrycja Dajnowicz-Brzezik, Patrycja Milewska, Paweł Knapp, Anna Zalewska, Adrian Chabowski

Table S1. List of primers used for real-time PCR in the study.

Target Gene	Accession number	Forward Primer (5′-3′)	Reverse Primer (5′-3′)	Amplicon Length [bp]
MMP1	NM_002421	ACAGCCCAGTACTTATTCCCTTTG	GGGCTTGAAGCTGCTTACGA	74
MMP2	NM_004530	AGCGAGTGGATGCCGCCTTTAA	CATTCCAGGCATCTGCGATGAG	138
MMP3	NM_002422	AAATCCTGATCTTTAAAGACAGGCAC	GAGATCAAATGCAATTCAGGTTCA	76
MMP7	NM_002423	TCGGAGGAGATGCTCACTTCGA	GGATCAGAGGAATGTCCCATACC	127
MMP8	NM_002424	CCAAGTGGGAACGCACTAACTTGA	TGGAGAATTGTCACCGTGATCTCTT	200
MMP9	NM_004994	GCACGACGTCTTCCAGTACC	CAGGATGTCATAGGTCACGTAGC	124
MMP10	NM_002425	CAAAAGAGGAGGACTCCAACA	TTCACATCCTTTTCGAGGTTG	76
MMP11	NM_005940	AAGAGGTTCGTGCTTTCTGG	CCATGGGAACCGAAGGAT	72
MMP12	NM_002426	TGCTGATGACATACGTGGCA	AGGATTTGGCAAGCGTTGG	70
MMP13	NM_002427	CCTTGATGCCATTACCAGTCTCC	AAACAGCTCCGCATCAACCTGC	97
MMP14	NM_004995	GGATGGACACAGAGAACTTCGTG	CGAGAGGTAGTTCTGGGTTGAG	118
TIMP1	NM_003254	TGTTGTTGCTGTGGCTGATAGC	TCTGGTGTCCCCACGAACTT	118
TIMP2	NM_003255	ACCCTCTGTGACTTCATCGTGC	GGAGATGTAGCACGGGATCATG	129
TIMP3	NM_000362	TACCGAGGCTTCACCAAGATGC	CATCTTGCCATCATAGACGCGAC	132
PEPD	NM_000285	GAGAACTCAGCCGTGCTACACT	AAGGAGCAGGTGATGTCGGAAG	131
POX/PRODH	NM_016335	AGAGTCAGCGATGACGGCTTCA	CTTGCTCCACAGCCATTTGGTG	127
PYCR1	NM_006907	AAGATGGCAGGCTTGTGGAGCA	CAGAGCATCCAGGGCTGTGAAA	134
PYCR2	NM_013328	TGCAAGCCAGACACATCGTGGT	GTGTTGGTCATGCAGCGAATCAC	115
PYCR3	NM_023078	GTGGAAGCTCAGCACATACTGG	CTTGGTGGCAAAGATGACGAGC	135
Nrf2	NM_006164	CCAAAAGGAGCAAGAGAAAGCC	GGCAACCTGGGAGTAGTTGG	133

Sirt1	NM_012238	TAGACACGCTGGAACAGGTTGC	CTCCTCGTACAGCTTCACAGTC	117
GPx	NM_002085	ACAAGAACGGCTGCGTGGTGAA	GCCACACACTTGTGGAGCTAGA	100
GSR	NM_000637	TGGCACTTGCGTGAATGTTG	CACATAGGCATCCCGCTTTTC	157
TNFα	NM_000594	CTGGGGCCTACAGCTTTGAT	GGCTCCGTGTCTCAAGGAAG	170
NF-κB	NM_003998	GCAGCACTACTTCTTGACCACC	TCTGCTCCTGAGCATTGACGTC	130
IκB	NM_020529	CAGCAGACTCCACTCCACTT	GAGAGGGGTATTTCCTCGAA	114
β-actin	NM_001101	AGTCGGTTGGAGCGAGCATC	GGACTTCCTGTAACAACGCATCTC	115

Table S2. The receiver operating characteristic (ROC) analysis in HGSOC patients for lymph node metastasis.

Parameter	AUC	p value	95% confidence interval	Cut-off	Sensitivity [%]	Specificity [%]
MMP11	0.7143	0.0495	0.5231 - 0.9055	> 1.488	73.33	71.43
IκB	0.7188	0.0417	0.5249 - 0.9126	< 0.6655	68.75	71.43

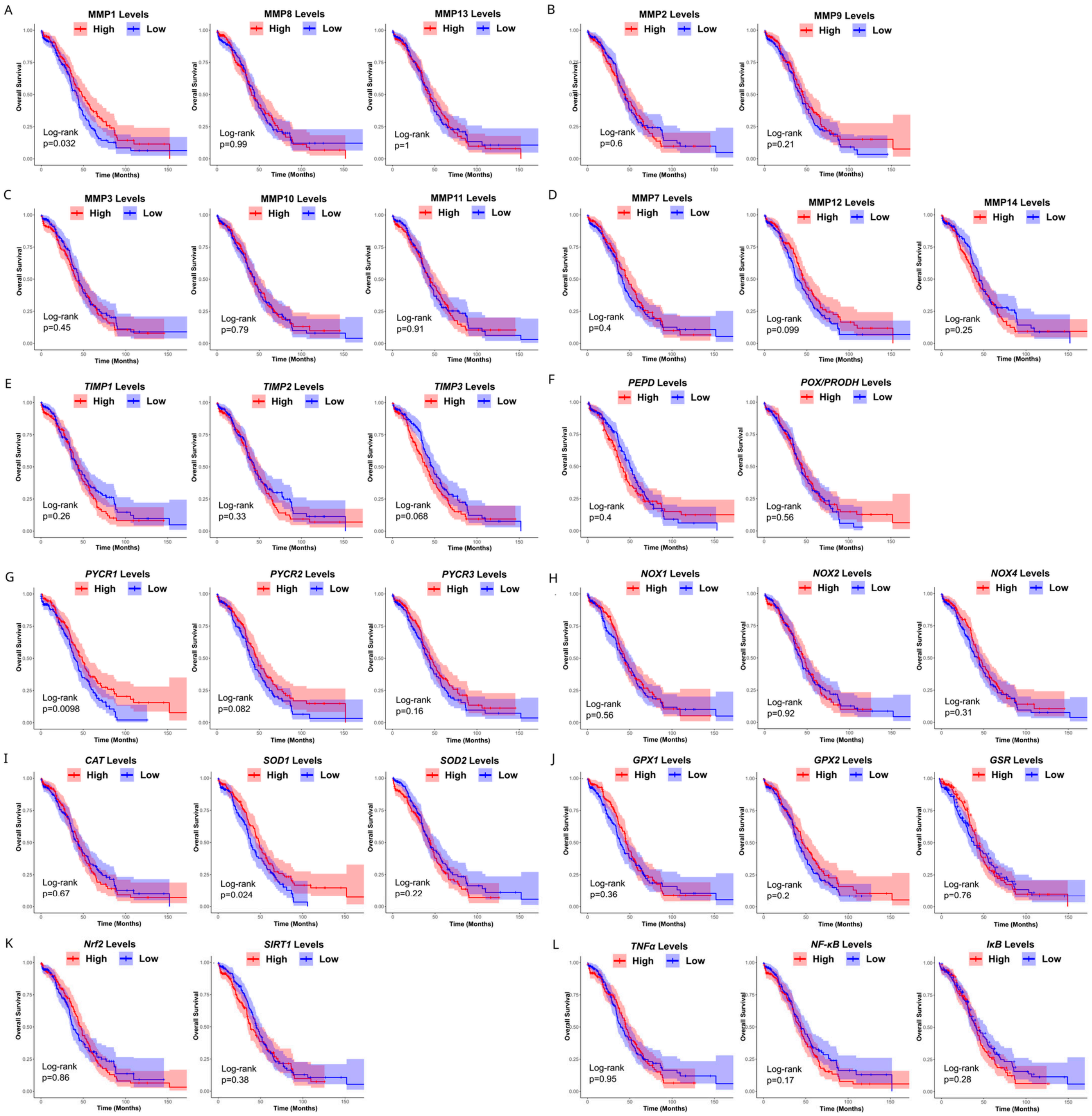

Figure S1. Kaplan-Meier overall survival curves of the matrix- (A-G) and oxidative stress-associated (H-L) genes based on TCGA dataset. OC samples were assigned into two separate groups depending on whether target expression of each sample is higher (high expression) or lower (low expression) than the median.

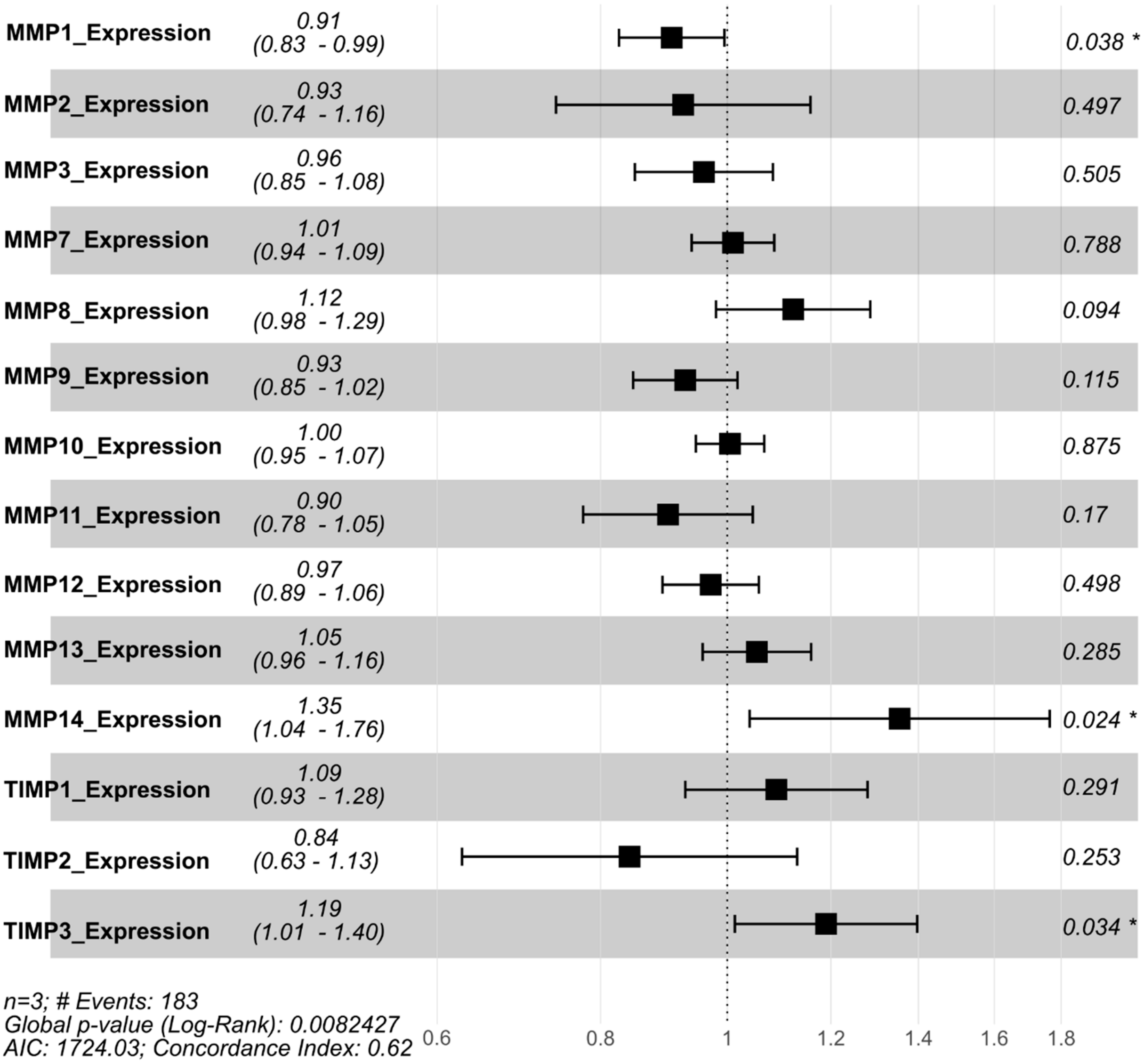

Figure S2. MMPs and TIMPs hazard ratio for overall survival in TCGA ovarian cancer patients. * p<0.05

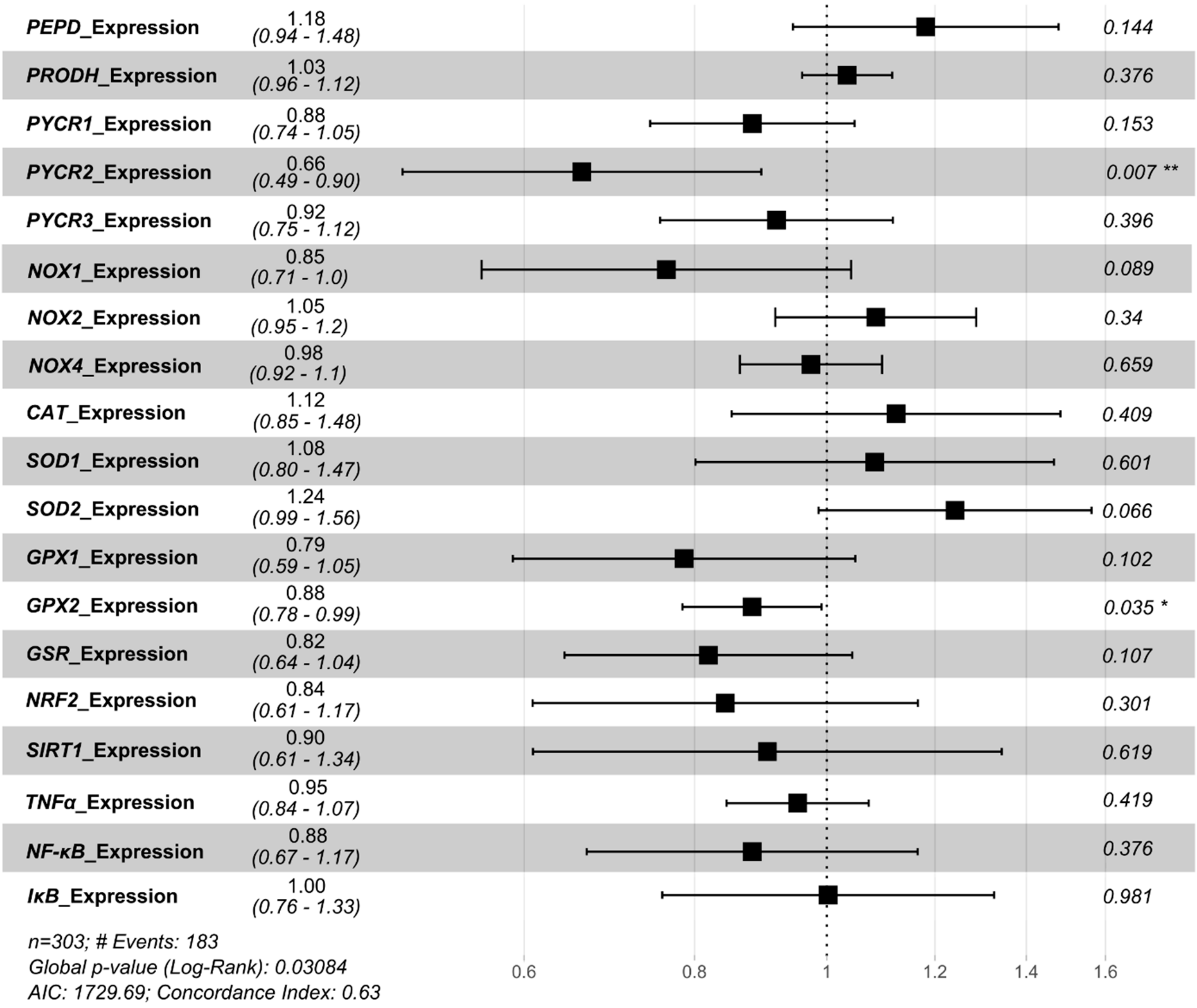

Figure S3. Oxidative stress-associated hazard ratio for overall survival in TCGA ovarian cancer patients. * $p<0.05$, ** $p<0.01$

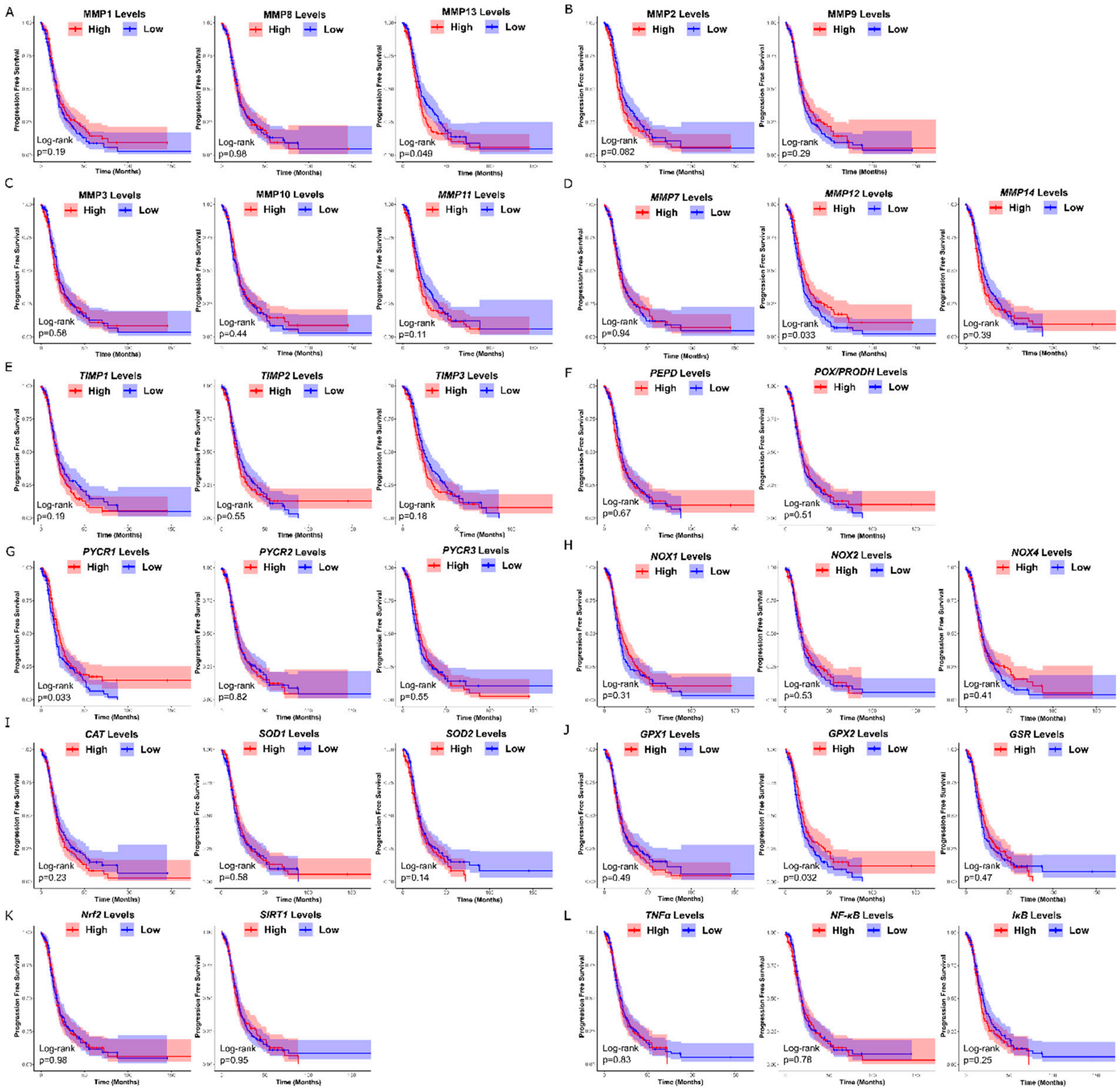

Figure S4. Kaplan-Meier progression free survival curves of the matrix- (A-G) and oxidative stress-associated (H-L) genes based on TCGA dataset. OC samples were assigned into two separate groups depending on whether target expression of each sample is higher (high expression) or lower (low expression) than the median.
